# Magnitude and Predictors of Antimicrobial Use-Related Drug Problems in Geriatric Inpatients: Evaluation of Pharmacist Interventions

**DOI:** 10.2174/0115748863427761251203131650

**Published:** 2026-03-16

**Authors:** Mian Waqar Hussain, Jisha Myalil Lucca, Shabaz Mohiuddin Gulam, Vineetha Menon

**Affiliations:** 1 Department of Pharmacy Practice, College of Pharmacy, Gulf Medical University, Ajman 4184, United Arab Emirates;; 2 Clinical Pharmacy Department, Thumbay University Hospital, Ajman 4184, United Arab Emirates

**Keywords:** Antimicrobial stewardship, drug-related problems, infection control, geriatrics, inpatients, pharmaceutical services, teaching hospital, United Arab Emirates

## Abstract

**Introduction:**

Infections remain a major cause of illness and death in older adults. Physiological changes, polypharmacy, and chronic conditions complicate antimicrobial therapy and increase the risk of drug problems. Pharmacist-led interventions improve prescribing, but evidence from the United Arab Emirates is limited. This study evaluated the magnitude and determinants of antimicrobial use-related drug problems and assessed clinical pharmacist interventions in hospitalized older adults with infections.

**Methods:**

A retrospective observational study was conducted from February to July 2022 at a 350-bed tertiary care academic hospital in the United Arab Emirates. Medical records of patients aged 60 years and older admitted with infections and treated with systemic antimicrobials were reviewed. Drug-related problems were classified using the Hepler and Strand framework.

**Results:**

Among 102 patients, 85 antimicrobial-related drug problems were identified, with half experiencing at least one. Common categories were drug-drug interactions (29%), unnecessary use (18%), and overdosing (14%). Cephalosporins (21%), penicillins (12%), and fluoroquinolones (12%) were the most frequently implicated classes. Clinical pharmacists made 79 recommendations; 95% were accepted, and 91% were implemented. Main interventions were drug omission (28%), substitution (25%), and dose modification (23%). Prior antimicrobial use, current antimicrobial burden, and longer hospital stay predicted drug problems.

**Discussion:**

The high prevalence of antimicrobial use-related drug problems highlights the challenges of prior antimicrobial exposure, concurrent antimicrobial polypharmacy, and prolonged hospitalization. Findings reinforce the feasibility and acceptance of pharmacist-led interventions, supporting their role in antimicrobial stewardship.

**Conclusion:**

Clinical pharmacists are integral to optimizing antimicrobial therapy and improving medication safety in older adults.

## 1 INTRODUCTION

Life expectancy is increasing worldwide, leading to a rapid rise in the aging population. According to the WHO, by 2030, one in six people globally will be aged 60 years or older. Advancing age is associated with complex medical conditions and greater vulnerability to infections [1]. Infections remain a leading cause of morbidity and mortality among older adults, [2-5] accounting for one-third of all deaths in those aged 65 years and above, with an annual risk of serious infection estimated at 8% [2, 3]. This susceptibility arises from immunosenescence, multiple chronic conditions, and age-related organ decline [2-5]. Infections not only cause morbidity but also worsen pre-existing illnesses and accelerate functional decline. The most frequently affected sites include the urinary tract, lower respiratory tract, skin and soft tissues, and intra-abdominal regions [2, 6-8]. Karakoç-Parlayan *et al*. reported that skin and soft tissue infections were the leading cause of hospital admissions in patients aged 65-79 years, while sepsis and urinary tract infections predominated in those aged 80 years and above [8]. Other studies have highlighted pneumonia and influenza as major causes of death in this population [3, 6].

Antimicrobial therapy remains the cornerstone of infection management, yet its rational use in older patients is challenged by physiological changes, cognitive and functional barriers, polypharmacy, comorbidities, and altered pharmacokinetics and pharmacodynamics. These factors increase the risk of antimicrobial use-related drug problems such as inappropriate selection, dosing errors, Adverse Drug Reactions (ADRs), and Drug-Drug Interactions (DDIs) [9-11]. All antimicrobial classes, including antibiotics, antifungals, antimycobacterials, and antivirals, can cause adverse effects that compromise organ function. Several studies illustrate this burden. In Ethiopia, an average of 1.90 Drug-Related Problems (DRPs) per elderly patient was reported, with 47.6% related to treatment effectiveness [12]. In Portugal, 484 DRPs were identified with a median of 15 per patient, most being Adverse Drug Events (ADEs) [13]. A retrospective database analysis of 484,351 ADEs showed a nonlinear relationship between age and adverse outcomes, peaking among patients aged 60-69 years. Sulfamethoxazole-trimethoprim, ciprofloxacin, and levofloxacin were frequently implicated, while sulfamethoxazole-trimethoprim, vancomycin, ciprofloxacin, and piperacillin-tazobactam ranked among the top 20 drugs linked to severe reactions [14, 15].

As in other areas of geriatric care, infection management benefits from collaborative, multidisciplinary teamwork. Clinical pharmacists play a vital role, as patient-centered pharmaceutical care reduces DRPs and healthcare costs. Silva *et al*. reported that pharmacist recommendations produced an annual cost saving of €3,950 per patient, while pharmacist-led interventions reduced ADEs in residential aged care facilities [13]. In the United Arab Emirates (UAE), major steps have been taken to combat Antimicrobial Resistance (AMR) through the National Strategy and Action Plan for Combatting Antimicrobial Resistance (NAP-AMR), which promotes public and professional awareness [16]. Globally, pharmacist-led Antimicrobial Stewardship Programs (ASPs) are well-recognized for improving prescribing and reducing resistance [17, 18]. However, despite studies assessing antimicrobial utilization in the UAE, [19, 20] limited evidence exists on antimicrobial use-related DRPs among geriatric inpatients and the impact of clinical pharmacist interventions. To address this gap, the present study evaluates the extent and types of antimicrobial use-related DRPs, examines the nature and acceptance of pharmacist interventions, and identifies predictors associated with these problems in hospitalized older adults with infectious diseases.

## 2 MATERIALS AND METHODS

### 2.1 Study Design and Setting

This retrospective observational study was conducted at a 350-bed private, academic tertiary care hospital in Ajman, UAE. The hospital provides comprehensive multidisciplinary healthcare services and serves a demographically diverse patient population. In 2020, clinical pharmacy services were established. Clinical pharmacists engage in integrated activities to identify DRPs, including medication reconciliation, first-dose verification, comprehensive chart review, participation in ward rounds, and patient education. For all identified DRPs, the corresponding pharmacist interventions and physicians’ responses to those interventions are documented in the clinical pharmacists’ intervention records.

### 2.2 Study Period and Population

Data were collected retrospectively over a six-month period, from February to July 2022. The dataset comprised elderly patients hospitalized between January 1 and December 31, 2021, with data retrieved from electronic medical records and the hospital information system. The study population comprised patients aged 60 years and older, of any gender, who had been admitted to the internal medicine department for a minimum duration of 24 hours. Eligible patients were those with a confirmed diagnosis of an infectious condition, verified through both clinical documentation and ICD-10 coding, and who had received at least one systemic antimicrobial for either therapeutic or prophylactic purposes. Patients were excluded if they had been transferred to palliative or hospice care, had incomplete medical records or missing documentation related to medications or diagnoses, or were readmitted with the same infectious diagnosis during the study period. In such cases, only the first admission was considered to prevent duplication and ensure analytical consistency.

### 2.3 Study Instrument and Variables

A structured data abstraction form was developed based on an extensive literature review and consultations with clinical experts in infectious diseases and clinical pharmacy. The tool was designed to systematically capture demographic, clinical, pharmacological, and intervention-related variables aligned with the study objectives. To assess clarity and feasibility, a pilot test was conducted on a random sample of ten medical records. Based on the feedback received, minor revisions were made to improve the instrument's completeness, consistency, and usability.

The final version of the form comprised three main categories. The first captured demographic and clinical characteristics, including age, gender, Body Mass Index (BMI), smoking and alcohol consumption status, and the number of diagnosed comorbid conditions. It also included data on the Charlson Comorbidity Index (CCI), the number of healthcare-associated infections diagnosed during hospitalization, presence of renal or hepatic impairment, Intensive Care Unit (ICU) admission, length of hospital stay, and any prior hospitalization within the preceding 30 days. The second category focused on pharmacotherapy-related data, particularly the number of medications and antimicrobials prescribed during the hospital stay. For each medication, details regarding the name, dose, route of administration, frequency, duration, and therapeutic indication were recorded. Information regarding antimicrobial exposure within the 30 days prior to admission was also collected. The third category documented clinical pharmacist interventions related to antimicrobial therapy. Each identified DRP was classified using Hepler and Strand’s framework. For each intervention, data were recorded on the type of pharmacist recommendation, the treating physician's acceptance status, and the implementation status. For accepted and implemented interventions, outcomes were also noted.

### 2.4 Outcomes

The primary outcome of this study was the identification and characterization of antimicrobial use-related DRPs among hospitalized geriatric patients. Secondary outcomes included evaluating clinical pharmacist interventions addressing these DRPs, their acceptance and implementation by physicians, and the clinical outcomes following implementation. Additionally, determinants of DRP occurrence were analyzed using multivariate logistic regression to identify significant predictors.

### 2.5 Data Collection Procedure

A systematic retrospective review of electronic medical records and the hospital information system was conducted to extract relevant patient data. All patient records were screened and assessed against the inclusion criteria, and data were collected for all eligible patients over the six-month study period. For each patient, sociodemographic variables such as age, gender, and BMI, along with lifestyle characteristics including smoking and alcohol consumption, were recorded. Clinical parameters, including CCI scores, presence of renal or hepatic impairment, number of diagnosed comorbidities, development of healthcare-associated infections, need for ICU admission, length of hospital stay, and history of prior hospitalization, were also collected. Medication-related data captured included the total number of prescribed drugs and systemic antimicrobials during hospitalization, as well as prior antimicrobial use within the preceding 30 days. Information related to antimicrobial use-related drug problems was obtained through a detailed review of clinical pharmacists’ intervention documentation, progress notes, and electronic prescribing modules. Each DRP was classified based on the framework. DRPs captured in this study included untreated indications, inappropriate drug choice, drug/therapeutic duplication, subtherapeutic dosing, overdosing, failure to receive drugs, drug use without indication, DDIs, and ADRs. Clinical pharmacist interventions were analyzed and categorized by recommendation type, level of intervention, acceptance and implementation by the medical team, and subsequent implementation outcomes. All data were entered into a structured database following standardized coding rules. Upon completion of data extraction, the dataset was cleaned, categorized, and validated to ensure accuracy and readiness for statistical analysis.

### 2.6 Data Interpretation and Statistical Analysis

The validated dataset was exported to IBM SPSS Statistics for Windows, Version 26.0 (IBM Corp., Armonk, NY, USA) for analysis. Descriptive statistics were used to summarize the data: continuous variables were reported as means and standard deviations, while categorical variables were presented as frequencies and percentages. To identify factors associated with the occurrence of DRPs, bivariate logistic regression analyses were first conducted. Variables with a *p*-value ≤0.25 were subsequently included in a multivariate logistic regression model. Adjusted Odds Ratios (AORs) and 95% Confidence Intervals (CIs) were calculated to determine the strength of association. A *p*-value <0.05 was considered statistically significant.

### 2.7 Reporting Guidelines

This study is reported in accordance with the Strengthening the Reporting of Observational Studies in Epidemiology (STROBE) guidelines.

### 2.8 Ethical Considerations

The study protocol was reviewed and approved by the Institutional Ethics Committee (Ethics Reference Number: IRB/COP/STD/30/Dec-2021). The research was conducted in accordance with the ethical principles outlined in the Declaration of Helsinki. Given the retrospective nature of the study and the use of de-identified data, the requirement for informed consent was waived in accordance with institutional and national ethical guidelines. Patient confidentiality was rigorously maintained throughout the study. No personal identifiers were recorded, and all data were stored in encrypted, password-protected electronic files accessible only to authorized members of the research team.

## 3 RESULTS

Fig. (**1**) depicts the patient selection process. Of 424 geriatric patients screened, 146 received systemic antimicrobials for infectious etiologies. A total of 44 patients were excluded, yielding 102 eligible patients.

Table **1** summarizes the baseline characteristics of the study population. Most patients were aged 60-69 years (74%) and were male (75%). The mean BMI was 25.62, with 48% classified as having elevated BMI. Regarding lifestyle behaviors, 18% were current smokers, and 7% reported current alcohol consumption. Half of the patients (51%) had five or more diagnosed medical conditions, with a mean of 5.08 diagnoses per patient. Among those with healthcare-associated infections (*n =* 80), the majority had one infection (60%). Renal impairment was present in 19% of patients and hepatic impairment in 8%. Based on the CCI, 21% were classified as high risk and 9% as very high risk for 12-month mortality. The average 10-year survival rate was 53%, with 25% of patients in the lowest survival bracket (0-20%). ICU admission was required for 19% of patients. The average hospital stay was 30.37 days, with 94% hospitalized for ≥11 days. Readmission within 30 days was reported in 43% of cases, and antimicrobial use in the 30 days before admission was noted in 12%. During hospitalization, 81% received ≥10 medications (Mean *=* 13.10), and 62% were prescribed three or more antimicrobials (Mean *=* 3.31). Regarding clinical outcomes, the majority recovered (69%).

Table **2** presents the incidence and distribution of antimicrobial use-related drug problems. A total of 85 DRPs were identified in 66 patients, resulting in an occurrence rate of 65% and an overall mean of 0.83 DRPs per patient and 1.29 DRPs per affected patient. The majority of affected patients (50%) experienced one DRP.

Table **3** presents the classification of 85 antimicrobial use-related drug problems identified in the study, based on Hepler and Strand’s framework. The most frequently observed category was DDIs (29%), followed by drug use without indication (18%) and overdose (14%).

Table **4** presents the specific drugs implicated in each DRP category. The most frequent category was DDI, most often involving levofloxacin (6 cases). Drug use without indication was commonly observed with ceftriaxone (3 cases). Meropenem was the drug most frequently associated with both overdose and subtherapeutic dosing. Failure to receive a drug was most often reported with ceftriaxone, while inappropriate drug choice was primarily linked to piperacillin-tazobactam. ADRs occurred most frequently with β-lactams.

The details of various drug classes associated with DRPs are listed in Table **5**. Analyses of antimicrobial drug classes involved in DRPs revealed that cephalosporins (21%) were the most frequently implicated, followed by penicillins and fluoroquinolones (12% each), carbapenems (11%), oxazolidinones (6%), and glycopeptides (5%).

Fig. (**2**) presents the five antimicrobials most frequently associated with DRPs among hospitalized geriatric patients. Ceftriaxone was the leading contributor with 16 DRP cases, followed by meropenem (11), piperacillin-tazobactam (9), levofloxacin (9), and linezolid (7).

Table **6** presents the types of recommendations made by clinical pharmacists to address identified DRPs. Clinical pharmacists made 79 recommendations to address DRPs. The most frequent type was drug deletion (28%), followed by drug substitution (25%) and dose modification (23%).

Fig. (**3**) illustrates the number of clinical pharmacists’ recommendations made, accepted, and implemented by physicians across various types of DRPs. A total of 79 recommendations were made, of which 75 were accepted and 72 implemented, yielding high acceptance (95%) and implementation (91%) rates.

Table **7** presents the outcomes of clinical pharmacists’ recommendations that were implemented. Among implemented interventions, drug withdrawal (28%), dose modification (24%), and drug substitution (22%) were the most common.

Table **8** summarizes the regression analyses of predictors associated with antimicrobial use-related problems. Prior antimicrobial exposure within the past 30 days was significantly associated with an increased risk (AOR: 4.96; 95% CI: 1.23–20.03; *p* = 0.025), indicating that geriatric patients with recent antimicrobial use were nearly 5 times more likely to develop a DRP during hospitalization compared to those without such exposure. The number of antimicrobials prescribed during hospitalization was also an independent predictor (AOR: 1.63; 95% CI: 1.20–2.22; *p* = 0.002), with each additional antimicrobial increasing the odds of a DRP by 63%. In addition, a longer hospital stay was associated with a higher risk (AOR: 1.03; 95% CI: 1.00–1.06; *p* = 0.026), reflecting a 3% increase in likelihood for each additional day of admission.

## 4 DISCUSSION

This study highlights important findings on antimicrobial use and DRPs in hospitalized elderly patients in the UAE. Most patients (81%) were taking ten or more medications, and 62% received three or more antimicrobials, increasing the likelihood of drug-related issues, given their multiple health conditions and organ dysfunction. A recent review reported a similar trend, showing polypharmacy among 25.3% of adults aged 65-74, 36.4% aged 75-84, and 46.5% aged ≥ 85 years, and linking it to negative outcomes such as frailty, longer hospital stays, and higher mortality [21]. Compared with global data [22-24], the longer mean hospital stay (30.4 days) and high rate of healthcare-associated infections (78%) in our cohort indicate a more complex clinical profile. Nevertheless, the relatively low in-hospital mortality rate (6%) may reflect the positive impact of clinical pharmacist interventions, consistent with international studies showing that pharmacist-led stewardship improves antimicrobial appropriateness, reduces adverse events, and enhances outcomes [12, 13]. Variations from global trends could be due to several factors, including the infection types, patient age, and differences in healthcare settings.

Among 102 geriatric patients (Mean age 68 years), 85 DRPs were identified, averaging 0.83 per patient. The most common problems were DDIs (29%), unnecessary antimicrobial use (18%), and overdosing (14%). Similar findings were reported by Ma *et al*., where 55.2% of elderly patients with chronic obstructive pulmonary disease exacerbations experienced DRPs (Mean 0.72 per patient) [25]. In contrast, Hailu *et al*. observed a higher DRP burden (Mean 1.9 per patient) [12], while Silva *et al*. identified 484 DRPs among 31 elderly patients (Median 15 per patient), primarily non-allergic ADEs, unnecessary treatments, and cost-related issues [13]. Differences in DRP incidence and patterns across these studies may reflect variations in patient age, comorbidity complexity, prescribing patterns, and the level of pharmacist involvement in care.

Analysis of antimicrobial classes implicated in DRPs revealed cephalosporins (21%) as the most frequent, followed by penicillins and fluoroquinolones (12% each), carbapenems (11%), oxazolidinones (6%), and glycopeptides (5%). This pattern aligns with previous reports highlighting β-lactams as major contributors to DRPs in hospitals. Shamna *et al*. found cephalosporins among antibiotics with the highest ADR risk scores [26]. Additionally, fluoroquinolones have been repeatedly linked to safety concerns in older adults, including significant DDIs and class-specific toxicities such as QT prolongation and tendinopathy [27].

Our study showed high pharmacist-physician collaboration, with 95% of pharmacist recommendations accepted and 91% implemented, reflecting the strong impact of clinical pharmacy services on antimicrobial optimization. Similar studies have reported comparable acceptance rates. A study by Hailu *et al*. reported a prescriber acceptance rate of 91.7% [12]. Similarly, an internal medicine-focused study reported a 95.6% acceptance rate for pharmacist-led antimicrobial stewardship interventions [28]. These consistently high rates underscore physicians’ confidence in pharmacist-driven interventions.

The most frequent pharmacist recommendations in our study were drug discontinuation (28%), substitution (25%), and dose adjustment (23%), indicating an emphasis on therapy rationalization and individualized dosing. In comparison, Ertuna *et al*. reported monitoring (31%), discontinuation (20%), and dose adjustments (14%) as the most frequent interventions [29]. While specific patterns varied, both studies affirm the proactive and comprehensive nature of pharmacists’ roles in identifying and resolving DRPs. Similarly, a randomized clinical trial in Quebec showed that 58% of physicians implemented pharmacists’ recommendations for discontinuation or substitution, demonstrating the practical influence of such interventions on prescribing practices [30]. Collectively, these findings reinforce the value of pharmacist involvement in enhancing the safety and effectiveness of antimicrobial therapy in complex geriatric cases.

Predictors of DRPs in this study included prior antimicrobial exposure, a higher number of prescribed antimicrobials during hospitalization, and longer hospital stays. These factors have been consistently associated with increased DRP risk in the literature. A *PLOS ONE* study of 272 surgical inpatients found prolonged hospitalization (AOR 3.57; *p < *0.001) and polypharmacy (≥5 medications; AOR 2.08; *p < *0.05) to be independent predictors of antibiotic use-related problems [31]. Similarly, multiple studies identified polypharmacy and multimorbidity as key contributors to higher DRP rates [12, 15, 29]. Yadesa *et al*. reported that prolonged hospital stay (>11 days) was associated with a threefold increase in antimicrobial use-related problems [32], while a study from Egypt found that prior antibiotic use within three months increased the risk of treatment complications in urinary tract infections [33]. These findings corroborate our results and reflect how cumulative exposure, extended hospitalization, and complex medication regimens amplify the likelihood of inappropriate prescribing, interactions, and adverse reactions in elderly patients.

## CONCLUSION

This study demonstrated a high prevalence of antimicrobial use-related DRPs among hospitalized older adults, with DDIs, unnecessary antimicrobial use, and overdosing being the most frequent. Clinical pharmacists’ recommendations were largely accepted and implemented, highlighting their contribution to optimizing antimicrobial therapy and enhancing medication safety in this population. These findings support the inclusion of pharmacists within multidisciplinary care teams and may inform local antimicrobial stewardship initiatives. Further prospective studies are warranted to assess the long-term impact of pharmacist-led interventions on clinical outcomes and healthcare utilization.

## STUDY LIMITATIONS

This study has some limitations that should be considered when interpreting the results. First, its retrospective observational design relied on the completeness and accuracy of documentation in electronic medical records and pharmacist intervention logs, which may have led to underreporting or misclassification of DRPs and interventions. Second, the study was conducted in a single tertiary care academic hospital, which may limit the generalizability of the findings to other healthcare settings, particularly primary care or smaller hospitals with different clinical pharmacy practices and prescribing patterns. Third, the study did not include a comparator group without pharmacist involvement, preventing assessment of the direct causal impact of interventions on patient outcomes. Finally, potential confounding factors, including infection severity, patient functional status, and prescribing behaviors of individual clinicians, were not fully captured, which may have influenced the occurrence of DRPs.

## AUTHORS' CONTRIBUTIONS

The authors confirm their contribution to the paper as follows: Study conception and design: V.M.; Data collection: M.W.H.; Analysis and interpretation of results: V.M., S.M.G.; Draft manuscript: V.M., J.M.L., M.W.H.; Final manuscript review and editing: V.M., J.M.L., S.M.G. All the authors reviewed and approved the final version of the manuscript.

## Figures and Tables

**Fig. (1):**
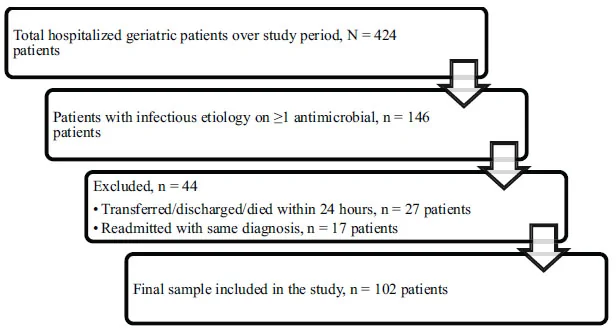
Flowchart of patient recruitment. **Note:** The figure illustrates the stepwise inclusion of patients into the study. A total of 424 hospitalized geriatric patients were screened during the study period. Of these, 146 patients with infectious etiology who received at least one antimicrobial were identified. Forty-four patients were excluded: 27 who were transferred, discharged, or died within 24 hours, and 17 who were readmitted with the same diagnosis. The final study sample included 102 patients.

**Fig. (2):**
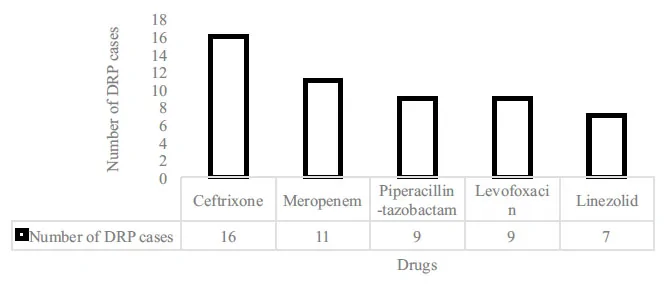
Top five antimicrobials contributing to DRPs. **Note:** The bar chart presents the distribution of drug-related problems across specific antimicrobials prescribed to hospitalized geriatric patients. The y-axis shows the number of DRP cases, and the x-axis lists the implicated drugs. Ceftriaxone was associated with the highest number of DRPs (*n =* 16), followed by meropenem (*n =* 11), piperacillin-tazobactam (*n =* 9), levofloxacin (*n =* 9), and linezolid (*n =* 7).

**Fig. (3):**
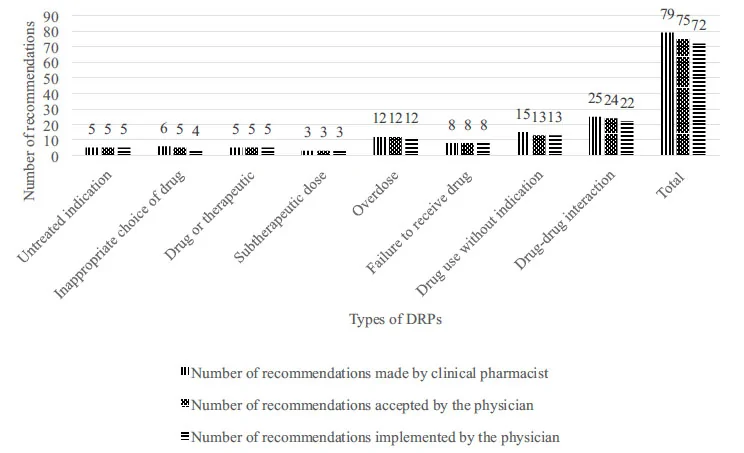
Acceptance and implementation rates of clinical pharmacists’ recommendations. **Note:** The bar chart illustrates the number of recommendations made by clinical pharmacists across different types of DRPs, alongside the number accepted and implemented by physicians. The x-axis shows the categories of DRPs, including untreated indication, inappropriate choice of drug, drug duplication, subtherapeutic dose, overdose, failure to receive drug, drug use without indication, and DDI. The y-axis indicates the number of recommendations. Overall, 79 recommendations were made by clinical pharmacists, of which 75 were accepted and 72 implemented. DDIs accounted for the highest number of recommendations (*n =* 25), followed by drug use without indication (*n =* 15) and overdose (*n =* 12).

**Table 1:** Sociodemographic and clinical characteristics of the study population (N = 102).

**Variables**	**Categories**	**Frequency (%)**
Age (YEARS)	60–69	75 (73.5)
	70–79	16 (15.7)
	≥80	11 (10.8)
	Mean (SD)	67.77 (7.92)
	Range	60–92
Gender	Male	77 (75.5)
	Female	25 (24.5)
BMI (m^2^)	<18.5	8 (7.8)
	18.5–24.9	45 (44.1)
	25.0–29.9	31 (30.4)
	≥30.0	18 (17.6)
	Mean (SD)	25.62 (6.14)
	Range	12.1–46.5
Smoking behavior	Current smokers	18 (17.6)
	Ex-smokers	3 (2.9)
	Never smokers	81 (79.4)
Drinking behavior	Current drinkers	7 (6.9)
	Abstainers	95 (93.1)
Number of diagnosed conditions	1–4	50 (49.0)
	≥5	52 (51.0)
	Mean (SD)	5.08 (3.06)
	Range	1–10
Number of healthcare-associated infections diagnosed following hospital admission (*n =* 80)	1	48 (60.0)
	2	29 (36.3)
	3	3 (3.8)
	Mean (SD)	1.12 (0.78)
	Range	1–5
Renal impairment	Yes	19 (18.6)
	No	83 (81.4)
Hepatic impairment	Yes	8 (7.8)
	No	94 (92.2)
CCI scoring system	12-month mortality rate Low Medium High Very high	34 (33.3) 38 (37.3) 21 (20.6) 9 (8.8)
	10-year survival rate 0–20 21–40 41–60 61–80 81–100 Mean (SD)	25 (24.5) 12 (11.8) 14 (13.7) 19 (18.6) 32 (31.4) 53.23 (36.96)
Required ICU care during hospital stay	Yes	19 (18.6)
	No	83 (81.4)
Length of hospital stay	≤ 10	6 (5.9)
	≥11	96 (94.1)
	Mean (SD)	30.37 (19.31)
	Range	7–93
Hospital admission in the past 30 days	Yes	44 (43.1)
	No	58 (56.9)
Antimicrobial use history in the last 30 days	Yes	12 (11.8)
	No	90 (88.2)
Number of medications prescribed during hospital stay	5–9	19 (18.6)
	≥ 10	83 (81.4)
	Mean (SD)	13.10 (3.78)
	Range	6–20
Number of prescribed antimicrobials during hospital stay	1	13 (12.7)
	2	26 (25.5)
	≥ 3	63 (61.8)
	Mean (SD)	3.31 (1.74)
	Range	1–7
Outcome of treatment	Recovered	70 (68.6)
	Dead	6 (5.9)
	Referred	23 (22.5)
	Discharged against medical advice	3 (2.9)

**Abbreviations:** BMI, Body Mass Index; CCI, Charlson Comorbidity Index; ICU, Intensive Care Unit; SD, Standard Deviation.

**Table 2:** Incidence and distribution of antimicrobial use-related problems (N = 102).

**Variables**	**Categories**	**Frequency (%)**
Number of DRPs per patient	No DRP	36 (35.3)
	One DRP	51 (50.0)
	Two DRPs	12 (11.8)
	Three DRPs	2 (2.0)
	Four DRPs	1 (1.0)
Total number of DRPs		85
Mean (SD)		0.83 (0.78)

**Abbreviations:** DRPs, Drug-Related Problems.

**Table 3:** Classification of antimicrobial use-related drug problems (n = 85).

**DRP Categories**	**Frequency (%)**
Untreated indication	5 (5.9)
Inappropriate choice of drug	6 (7.1)
Drug or therapeutic duplication	5 (5.9)
Subtherapeutic dose	3 (3.5)
Overdose	12 (14.1)
Failure to receive the drug	8 (9.4)
Drug use without indication	15 (17.6)
DDIs	25 (29.4)
Adverse drug reaction	6 (7.1)

**Abbreviations**: DDIs, Drug-Drug Interactions; DRPs, Drug-Related Problems.

**Table 4:** Drugs involved in antimicrobial use-related drug problems.

**DRP Categories**	**Drugs**
Inappropriate choice of drug	Cefepime (1), Ceftriaxone (2), Colistimethate (1), Piperacillin-tazobactam (2)
Drug or therapeutic duplication	Cefepime (1), Ceftriaxone (3), Cefuroxime (2), Ciprofloxacin (1), Ertapenem (1), Levofloxacin (1), Meropenem (1)
Subtherapeutic dose	Meropenem (2), Piperacillin-tazobactam (1)
Overdose	Cefepime (1), Ceftriaxone (1), Flucloxacillin (1), Linezolid (2), Meropenem (3), Piperacillin-tazobactam (4)
Failure to receive the drug	Ceftriaxone (5), Isoniazid (1), Meropenem (2)
Drug use without indication	Amoxicillin-clavulanic acid (2), Ceftriaxone (3), Cefuroxime (1), Colistimethate (1), Levofloxacin (1), Meropenem (3), Metronidazole (1), Piperacillin-tazobactam (1), Tigecycline (1), Vancomycin (1)
DDI	Amikacin (4), Amiodarone (2), Ceftriaxone (1), Chlorpromazine (1), Ciprofloxacin (3), Digoxin (1), Dolutegravir (1), Enoxaparin (1), Erythromycin (3), Escitalopram (1), Fentanyl (1), Ferrous gluconate (2), Ferrous sulfate (2), Fluconazole (1), Fludrocortisone (1), Fondaparinux (1), Furosemide (1), Levofloxacin (6), Linezolid (5), Methotrexate (1), Metoclopramide (1), Norepinephrine (1), Ondansetron (1), Rifampicin (1), Sodium alginate/Sodium bicarbonate/Calcium carbonate (1), Sodium citrate (1), Sulfamethoxazole-trimethoprim (1), Teicoplanin (2), Tramadol (1), Vancomycin (1), Warfarin (1)
Adverse drug reaction	Amoxicillin-clavulanic acid (1), Ceftriaxone (1), Cefuroxime (1), Levofloxacin (1), Piperacillin-tazobactam (1), Vancomycin (1)

**Notes:** Each drug may be involved in more than one category of DRP. **Abbreviations:** DDIs, Drug-Drug Interactions; DRPs, Drug-Related Problems.

**Table 5:** Drugs involved in DRPs by therapeutic class (n = 111).

**Therapeutic Classes**	**Drugs**	**ATC Code**	**Number of Cases**	**Total (%)**
Antacids, other combinations	Sodium alginate, Sodium bicarbonate, Calcium carbonate	A02BX13	1	1 (0.9)
Propulsives	Metoclopramide	A03FA01	1	1 (0.9)
Serotonin antagonists	Ondansetron	A04AA01	1	1 (0.9)
Vitamin K antagonists	Warfarin	B01AA03	1	1 (0.9)
Heparin group	Enoxaparin	B01AB05	1	1 (0.9)
Other antithrombotic agents	Fondaparinux	B01AX05	1	1 (0.9)
Iron bivalents, oral preparations	Ferrous gluconate	B03AA03	2	4 (3.6)
	Ferrous sulfate	B03AA07	2	
Salt solutions	Sodium citrate	B05CB02	1	1 (0.9)
Digitalis glycosides	Digoxin	C01AA05	1	1 (0.9)
Antiarrhythmics, class III	Amiodarone	C01BD01	2	2 (1.8)
Adrenergic and dopaminergic agents	Norepinephrine	C01CA03	1	1 (0.9)
High-ceiling diuretics and potassium-sparing agents	Furosemide	C03EB01	1	1 (0.9)
Mineralocorticoids	Fludrocortisone	H02AA02	1	1 (0.9)
Tetracyclines	Tigecycline	J01AA12	1	1 (0.9)
Penicillins	Flucloxacillin	J01CF05	1	13 (11.7)
	Amoxicillin-clavulanic acid	J01CR02	3	
	Piperacillin-tazobactam	J01CR05	9	
Cephalosporins	Cefuroxime	J01DC02	4	23 (20.7)
	Ceftriaxone	J01DD04	16	
	Cefepime	J01DE01	3	
Carbapenems	Meropenem	J01DH02	11	12 (10.8)
	Ertapenem	J01DH03	1	
Sulphonamides	Sulfamethoxazole-trimethoprim	J01EE01	1	1 (0.9)
Macrolides	Erythromycin	J01FA01	3	3 (2.7)
Aminoglycosides	Amikacin	J01GB06	4	4 (3.6)
Fluoroquinolones	Ciprofloxacin	J01MA02	4	13 (11.7)
	Levofloxacin	J01MA12	9	
Glycopeptides	Vancomycin	J01XA01	3	5 (4.5)
	Teicoplanin	J01XA02	2	
Polymyxins	Colistimethate	J01XB01	2	2 (1.8)
Imidazole derivatives	Metronidazole	J01XD01	1	1 (0.9)
Oxazolidinones	Linezolid	J01XX08	7	7 (6.3)
Antimycotics	Fluconazole	J02AC01	1	1 (0.9)
Antimycobacterials	Rifampicin	J04AB02	1	2 (1.8)
	Isoniazid	J04AC01	1	
Antivirals	Dolutegravir	J05AJ03	1	1 (0.9)
Other immunosuppressants	Methotrexate	L04AX03	1	1 (0.9)
Phenylpiperidine derivatives	Fentanyl	N02AB03	1	1 (0.9)
Other opioids	Tramadol	N02AX02	1	1 (0.9)
Phenothiazines	Chlorpromazine	N05AA01	1	1 (0.9)
Selective serotonin reuptake inhibitors	Escitalopram	N06AB10	1	1 (0.9)

**Abbreviations:** ATC, Anatomical Therapeutic Chemical classification system.

**Table 6:** Recommendations provided by clinical pharmacists (n = 79).

**Clinical Pharmacists’ Recommendations**	**Frequency (%)**
Addition of a drug	5 (6.3)
Deletion of the drug	22 (27.8)
Substitution of the drug	20 (25.3)
Change in schedule	6 (7.6)
Change in dose	18 (22.8)
Healthcare provider/patient education	8 (10.1)

**Table 7:** Outcomes of clinical pharmacists’ recommendations (n = 72).

**Outcome of Interventions**	**Frequency (%)**
Drug added	5 (6.9)
Drug withdrawn	20 (27.8)
Drug substituted	16 (22.2)
Change in administration frequency	6 (8.3)
Change in drug dose	17 (23.6)
Drug adherence ensured	8 (11.1)

**Table 8:** Determinants of antimicrobial use-related problems.

**Predictors**	**Bivariate Analysis**			**Multivariate Analysis**		
	**B**	**COR (95% CI)**	***p-*value**	**B**	**AOR (95% CI)**	***p-*value**
Age	-0.01	0.99 (0.95–1.05)	0.831			
Gender	0.03	1.03 (1.00–1.05)	0.047^*^			
BMI	-0.03	0.97 (0.91–1.04)	0.437			
Smoking behavior	0.19	1.21 (0.42–3.45)	0.725			
Drinking behavior	-1.25	0.29 (0.03–2.47)	0.255			
Number of diagnosed conditions	0.11	1.12 (0.97–1.29)	0.136			
Number of healthcare-associated infections diagnosed following hospital admission	0.41	1.51 (0.88–2.59)	0.139			
Renal impairment	-0.20	0.82 (0.28–2.37)	0.707			
Hepatic impairment	-0.82	0.44 (0.05–4.12)	0.474			
CCI scoring system						
12-month mortality rate						
Low		1 (Reference)				
Medium	0.34	0.71 (0.26–1.98)	0.518			
High	0.08	1.08 (0.39–3.01)	0.878			
10-year survival rate	-0.01	1.00 (0.98–1.01)	0.345			
Required ICU care during hospital stay	-0.86	0.43 (0.13–1.40)	0.158			
Length of hospital stay	0.03	1.03 (1.00–1.05)	0.047^*^	0.03	1.03 (1.00–1.06)	0.026^*^
Hospital admission in the past 30 days	-0.63	0.53 (0.23–1.24)	0.142			
Antimicrobial use history in the last 30 days	1.49	4.43 (1.23–15.94)	0.023^*^	1.60	4.96 (1.23–20.03)	0.025^*^
Number of medications prescribed during hospital stay	0.16	1.18 (1.04–1.33)	0.009^*^			
Number of prescribed antimicrobials during hospital stay	0.42	1.52 (1.14–2.02)	0.004^*^	0.49	1.63 (1.20–2.22)	0.002^*^

**Notes:** ^*^ *p*-value <0.05 was considered statistically significant. **Abbreviations:** AOR, Adjusted Odds Ratio; B, Regression Coefficient; CCI, Charlson Comorbidity Index; CI, Confidence Interval; COR, Crude Odds Ratio.

## Data Availability

The data supporting the findings of this article will be made available by the corresponding author upon reasonable request.

## LIST OF ABBREVIATIONS

ADEs: Adverse Drug Events
ADRs: Adverse Drug Reactions
AMR: Antimicrobial Resistance
AOR: Adjusted Odds Ratio
ASPs: Antimicrobial Stewardship Programs
ATC: Anatomical Therapeutic Chemical
BMI: Body Mass Index
CCI: Charlson Comorbidity Index
CI: Confidence Interval
COR: Crude Odds Ratio
DDIs: Drug-Drug Interactions
DRPs: Drug-Related Problems
ICU: Intensive Care Unit
ICD-10: International Classification of Diseases, Tenth Revision
NAP-AMR: National Strategy and Action Plan for Combating Antimicrobial Resistance
SD: Standard Deviation
UAE: United Arab Emirates
WHO: World Health Organization

## STANDARDS OF REPORTING

STROBE guidelines were followed.

## References

[1] World Health Organization (2025). World Health Organization. Ageing and health. Geneva: World Health Organization.

[2] Struyf T., Nuyts S., Tournoy J., Vaes B., Verbakel J.Y., Van den Bruel A. (2021). Burden of infections on older patients presenting to general practice: A registry-based study.. Fam Pract.

[3] Esme M., Topeli A., Yavuz B.B., Akova M. (2019). Infections in the elderly critically-ill patients.. Front Med (Lausanne).

[4] Mancinetti F., Marinelli A., Boccardi V., Mecocci P. (2024). Challenges of infectious diseases in older adults: From immunosenescence and inflammaging through antibiotic resistance to management strategies.. Mech Ageing Dev.

[5] Dimopoulos G., Koulenti D., Blot S., Extended Prevalence of Infection in Intensive Care Study Investigators (2013). Extended Prevalence of Infection in Intensive Care Study Investigators. Critically ill elderly adults with infection: Analysis of the extended prevalence of infection in intensive care study.. J Am Geriatr Soc.

[6] Debonera F. (2021). Infections in older adults: The art of early recognition.. Ann Longterm Care.

[7] Centers for Disease Control and Prevention (2024). People at increased risk for severe respiratory illnesses.. https://www.cdc.gov/respiratory-viruses/risk-factors/older-adults.html.

[8] Karakoç Parlayan H.N., Bulut D., Haykir Solay A., Kuzi S., Arikan T., Şencan I. (2025). An analysis of bacterial infection distribution in the elderly and their clinical and laboratory profiles.. Infect Diseas Clin Microbiol.

[9] Soraci L., Cherubini A., Paoletti L. (2023). Safety and tolerability of antimicrobial agents in the older patient.. Drugs Aging.

[10] Alves J., Prendki V., Chedid M., Yahav D., Bosetti D., Rello J. (2024). Challenges of antimicrobial stewardship among older adults.. Eur J Intern Med.

[11] Biedron C., Chopra T. (2013). Issues surrounding antibiotic use in older adults.. Curr Transl Geriatr Exp Gerontol Rep.

[12] Hailu B.Y., Berhe D.F., Gudina E.K., Gidey K., Getachew M. (2020). Drug related problems in admitted geriatric patients: The impact of clinical pharmacist interventions.. BMC Geriatr.

[13] Silva C., Ramalho C., Luz I., Monteiro J., Fresco P. (2015). Drug-related problems in institutionalized, polymedicated elderly patients: Opportunities for pharmacist intervention.. Int J Clin Pharm.

[14] Zagaria M.A.E. (2019). Addressing challenges of infection: Antibiotic adverse drug events in older adults.. US Pharm.

[15] Moore V.R., Glassman P.A., Au A., Good C.B., Leadholm T.C., Cunningham F.E. (2019). Adverse drug reactions in the Veterans Affairs healthcare system: Frequency, severity, and causative medications analyzed by patient age.. Am J Health Syst Pharm.

[16] Ministry of Health and Prevention (MOHAP). National Strategy and Action Plan for Combatting Antimicrobial Resistance (NAPAMR). United Arab Emirates: MOHAP. 20205.. https://mohap.gov.ae/en/w/national-strategy-and-action-plan-for-combatting-antimicrobial-resistance-nap-amr.

[17] Togoobaatar G., Ikeda N., Ali M. (2010). Survey of non-prescribed use of antibiotics for children in an urban community in Mongolia.. Bull World Health Organ.

[18] McKinn S., Trinh D.H., Drabarek D. (2021). Drivers of antibiotic use in Vietnam: Implications for designing community interventions.. BMJ Glob Health.

[19] Abdulrazzaq N., Chkhis A. (2024). Prevalence of antimicrobial use and healthcare-associated infections in the UAE: Results from the first nationwide point-prevalence survey.. Infect Dis Now.

[20] Thomsen J., Abdulrazzaq N.M., AlRand H. (2024). The UAE AMR Surveillance Consortium. Epidemiology and antimicrobial resistance trends of Acinetobacter species in the United Arab Emirates: A retrospective analysis of 12 years of national AMR surveillance data.. Front Public Health.

[21] Pazan F., Wehling M. (2021). Polypharmacy in older adults: A narrative review of definitions, epidemiology and consequences.. Eur Geriatr Med.

[22] Hoffman T., Margalit I., Tabah A. (2025). Risk factors for mortality among older adults with hospital-acquired bloodstream infections in the intensive care unit: A multicenter cohort study.. Infect Dis Ther.

[23] Moradi S., Najafpour Z., Cheraghian B., Keliddar I., Mombeyni R. (2024). The extra length of stay, costs, and mortality associated with healthcare-associated infections: A case-control study.. Health Sci Rep.

[24] Guillon A., Hermetet C., Barker K.A. (2020). Long-term survival of elderly patients after intensive care unit admission for acute respiratory infection: A population-based, propensity score-matched cohort study.. Crit Care.

[25] Ma Z., Shi H., Hong G., Wu L., Lin Y. (2025). Drug-related problems in elderly patients with AECOPD and pharmaceutical intervention practice: A prospective study.. Front Pharmacol.

[26] Shamna M., Dilip C., Ajmal M. (2014). A prospective study on adverse drug reactions of antibiotics in a tertiary care hospital.. Saudi Pharm J.

[27] Owens R.C. (2008). An overview of harms associated with β-lactam antimicrobials: Where do the carbapenems fit in?. Crit Care.

[28] Rizzo A., Suchindran S., Albrecht B., Metzger N.L. (2024). Impact of internal medicine pharmacists on antimicrobial stewardship.. J Am Coll Clin Pharm.

[29] Ertuna E., Arun M.Z., Ay S., Fatma Özge Kayhan Koçak FÖ, Gökdemir B., İspirli G. (2019). Evaluation of pharmacist interventions and commonly used medications in the geriatric ward of a teaching hospital in Turkey: A retrospective study.. Clin Interv Aging.

[30] Martin P., Tamblyn R., Benedetti A., Ahmed S., Tannenbaum C. (2018). Effect of a pharmacist-led educational intervention on inappropriate medication prescriptions in older adults: The D-PRESCRIBE randomized clinical trial.. JAMA.

[31] Niriayo Y.L., Ayalneh M., Demoz G.T., Tesfay N., Gidey K. (2023). Antimicrobial use related problems and determinants in surgical ward of Ethiopian tertiary hospital.. PLoS One.

[32] Yadesa T.M., Gudina E.K., Angamo M.T. (2015). Antimicrobial use-related problems and predictors among hospitalized medical inpatients in Southwest Ethiopia: A prospective observational study.. PLoS One.

[33] Shaker M., Zaki A., Asser S.L., Sayed I.E. (2024). Trends and predictors of antimicrobial resistance among patients with urinary tract infections at a tertiary hospital facility in Alexandria, Egypt: A retrospective record-based classification and regression tree analysis.. BMC Infect Dis.

